# Combined Naviculocuneiform and Calcaneocuboid Dislocation After Low-Energy Blunt Trauma: A Case Report

**DOI:** 10.7759/cureus.76319

**Published:** 2024-12-24

**Authors:** Megan Audet, Paul Fortin, Allan Grant, Thomas Stuut, Graham Englert

**Affiliations:** 1 Orthopaedic Surgery, Corewell Health William Beaumont University Hospital, Royal Oak, USA

**Keywords:** calcaneocuboid dislocation, midfoot injury, navicular fracture, naviculocuneiform dislocation, orif navicular

## Abstract

We present a case of a unique midfoot injury pattern including dislocations of the calcaneocuboid and naviculocuneiform joints with associated calcaneus and navicular fractures after low-energy injury. This combination of injuries is rare, especially with a low-energy mechanism. There are no current treatment guidelines. In this case, the patient was treated with open reduction and internal fixation of the navicular fracture, along with bridge plating of the calcaneocuboid joint, which was subsequently removed nine weeks post-operatively with good functional outcomes.

## Introduction

Midfoot injuries are uncommon, accounting for only 5% of foot trauma [1]. Fracture dislocations of the Chopart and Lisfranc joints are the most commonly suffered injuries of the midfoot [1]; however, combined dislocations of the calcaneocuboid and naviculocuneiform joints have been rarely described [2-8]. There are no current reports of this injury pattern with an associated navicular fracture. This case report describes the initial assessment, diagnosis, and management of a patient with calcaneocuboid and naviculocuneiform dislocations, accompanied by a calcaneus fracture extending into the calcaneocuboid articulation - an exceedingly rare injury pattern.

## Case presentation

**Figure 1:**
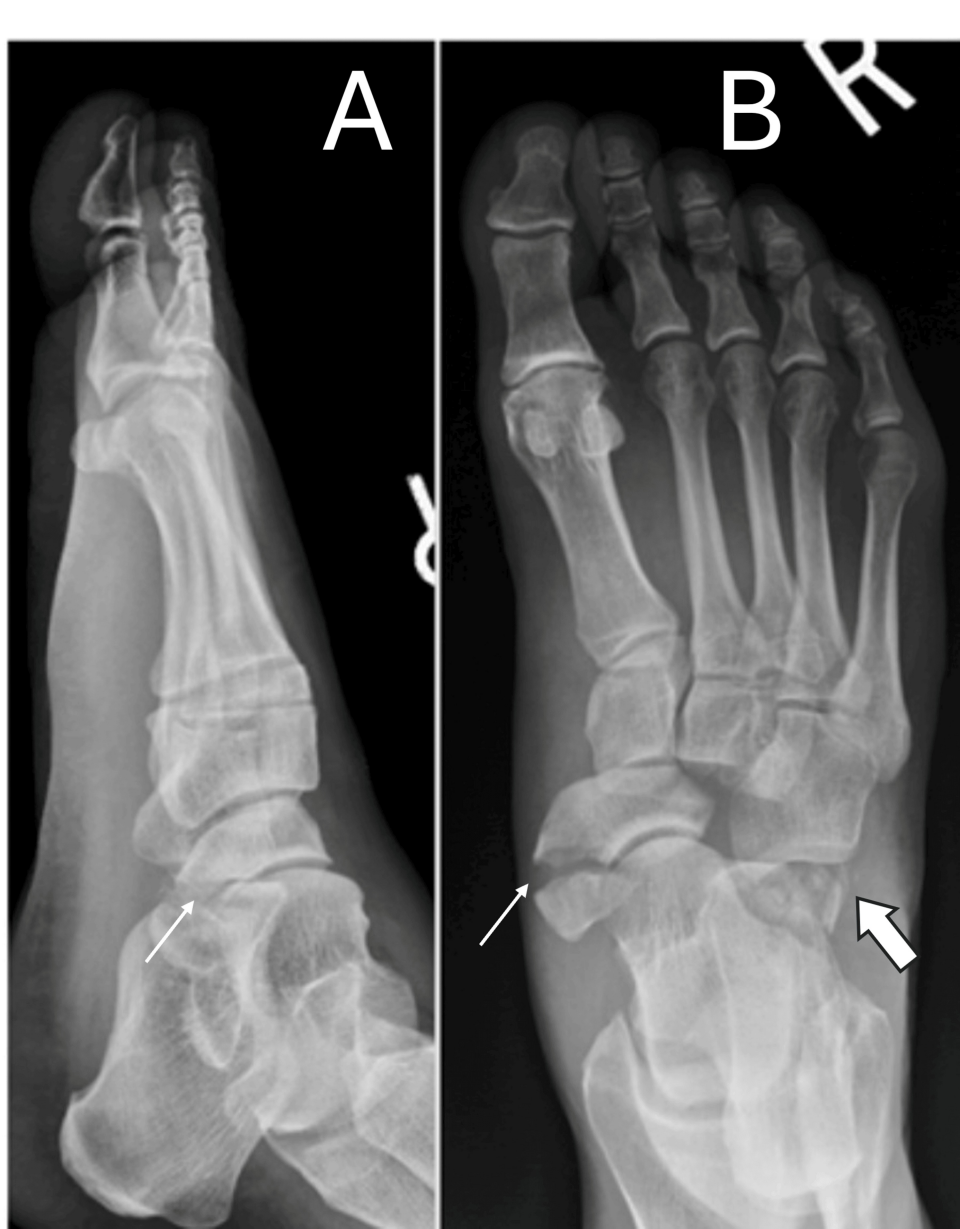
AP (B) and lateral (A) radiographs demonstrating naviculocuneiform dislocation with associated navicular fracture (small arrow (B)). Calcaneocuboid dislocation with a fracture of the anterior process of the calcaneus (small arrow (A), large arrow (B)).

**Figure 2:**
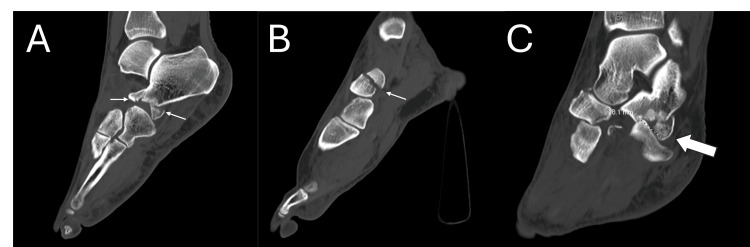
Selected sagittal (A, B) and coronal (C) cuts from preoperative CT imaging demonstrating naviculocuneiform dislocation with navicular fracture (small arrow (B)) and calcaneocuboid dislocation with fractures of the anterior process of the calcaneus (small arrows (A); large arrow (C)).

A 47-year-old female patient presented to the emergency department with right foot pain after a fall down three stairs. Upon examination, she had swelling and ecchymosis throughout the foot with severe tenderness along the midfoot and a palpable bony prominence medially. Radiographs of the foot revealed naviculocuneiform and calcaneocuboid dislocations with associated navicular fracture and fracture of the anterior process of the calcaneus (Figure 1). A closed reduction under conscious sedation was attempted in the emergency department but was unsuccessful. A CT scan was obtained, which re-demonstrated this injury pattern (Figure 2). The patient was admitted for surgery the following morning.

**Figure 3:**
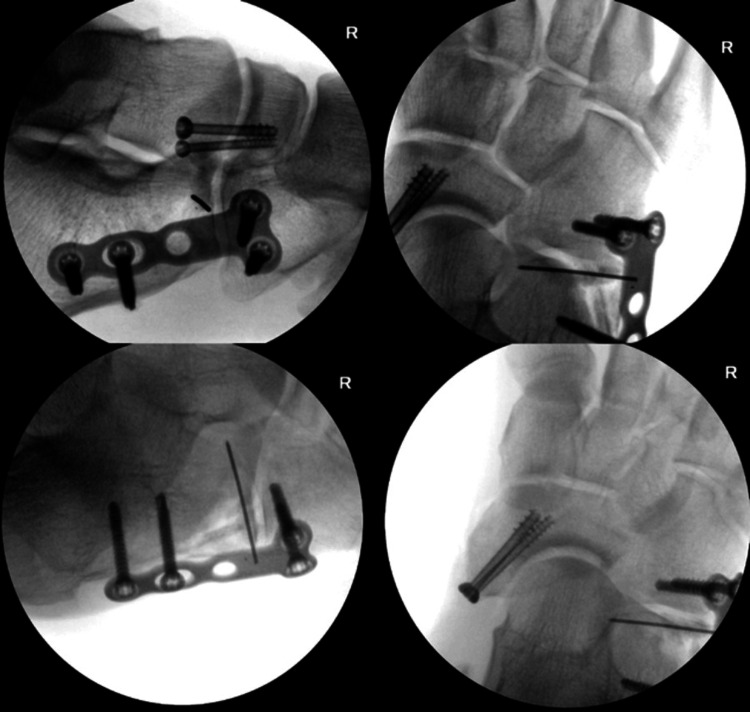
Intra-operative fluoroscopy demonstrating repair of the navicular fracture with two partially threaded cannulated screws. The articular surface of the calcaneus was reduced with a K-wire, and the calcaneocuboid joint was stabilized with a spanning plate. K-wire, Kirschner wire

The patient was brought to the operating room and positioned supine with a bone foam leg elevator. A dorsomedial approach was made to the navicular bone and the medial cuneiform was found to be dislocated laterally. The navicular fracture was reduced with one 3.0 mm and one 4.0 mm partially-threaded cannulated screws. Reduction of the naviculocuneiform joint was attempted at this time but was unsuccessful. A second dorsolateral incision was made over the calcaneocuboid joint. The cuboid was found to be impacted into the lateral and superior aspects of the calcaneus, causing a comminuted fracture of the articular surface of the anterior process of the calcaneus. The cuboid was levered out of its impacted position, and the fracture was reduced and held in position with a provisional percutaneous Kirschner wire (K-wire) from the cuboid into the calcaneus. This technique successfully reduced both the calcaneocuboid and naviculocuneiform joints. The lateral wall and calcaneocuboid articular fragments were fixed to the medial body of the articular surface of the calcaneus with a K-wire. A 3.5 mm five-hole Lapidus plate was used to span the calcaneocuboid joint and allow for the removal of the provisional pin spanning the joint (Figure 3). The wounds were copiously irrigated, and the incisions were closed in a layered fashion. The patient was placed in a bulky short-leg cast and was discharged home on post-operative day one.

**Figure 4:**
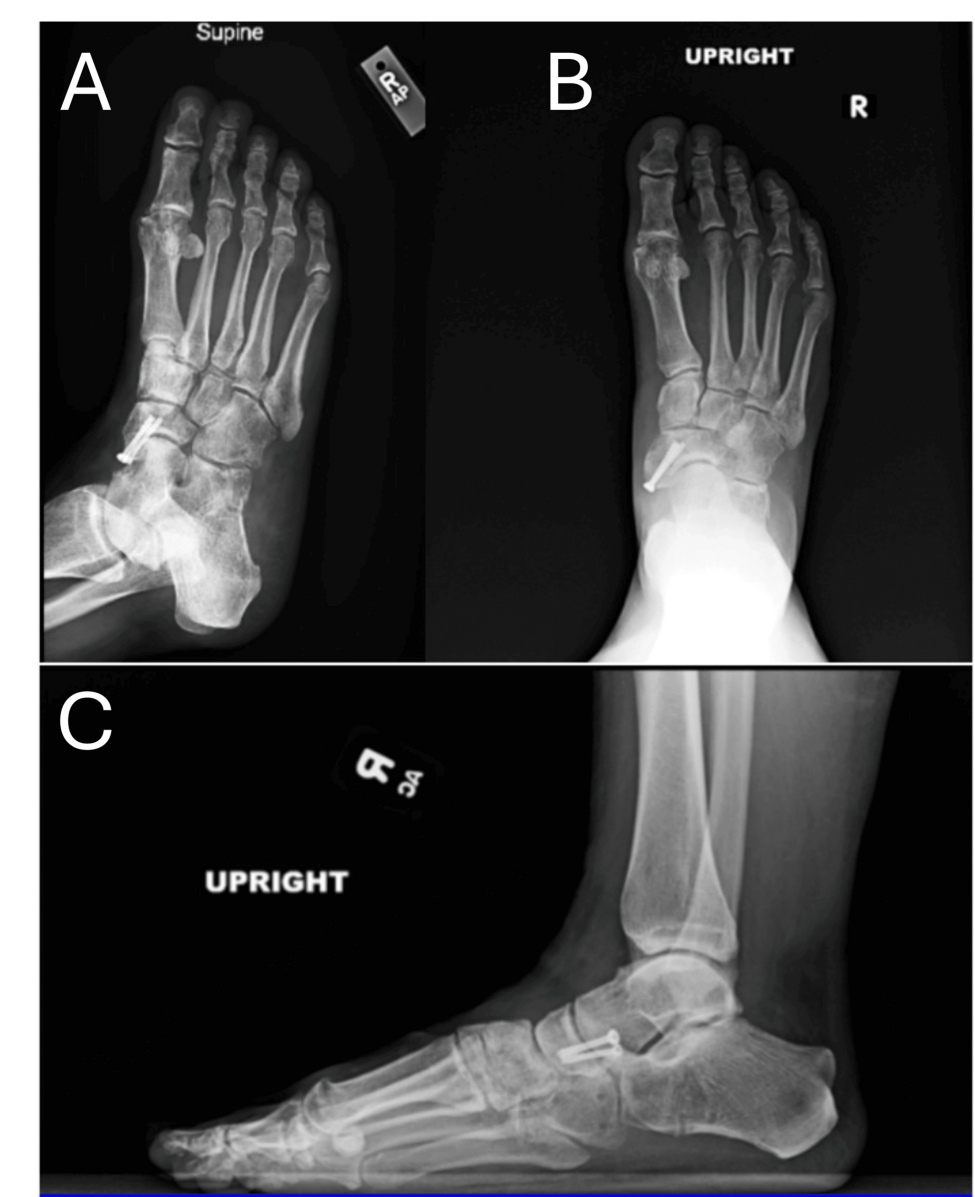
Standing AP (B), lateral (C), and oblique (A) radiographs obtained at four-month follow-up after elective removal of K-wire and spanning calcaneocuboid joint. K-wire, Kirschner wire

Six weeks post-operatively, the patient was transitioned to an aircast boot and instructed to remain non-weight bearing. At post-operative week nine, she underwent elective removal of the remaining K-wire and spanning plate across the calcaneocuboid joint. Three months following the index procedure, the patient began progressive weight bearing in the aircast boot. Four and a half months post-operatively, she was transitioned into a supportive shoe and returned to work (Figure 4).

## Discussion

Combined dislocations of the calcaneocuboid and naviculocuneiform joints are an extremely rare midfoot injury, which has been infrequently described in the literature [2,7]. The low incidence of these injuries is attributable to the strong dorsal and volar ligamentous attachments and rigid bony support of the midfoot. Good outcomes have been previously reported with both open reduction and internal fixation as well as open reduction and percutaneous pinning [1-3,6-8].

These injuries often occur following high-energy mechanisms such as motor vehicle collisions or crush injuries [1-3,6-8]. To our knowledge, there is only one prior report of combined calcaneocuboid and naviculocuneiform dislocation after low-energy injury. Dhole et al. presented a case of a 30-year-old female with this injury after a two-foot fall off of a stool, who was treated with open reduction and fixation with K-wires [5]. Our case represents a unique description of this injury pattern after a relatively low-energy mechanism of a fall down three stairs.

Furthermore, our case is a unique variation of this injury pattern due to the associated navicular fracture. We hypothesize that the fracture was a result of a continuation of abduction force through the naviculocuneiform joint. This fracture involved a significant portion of the talonavicular articular surface and required open reduction and internal fixation with cannulated screws. To our knowledge, this report is the first described case of calcaneocuboid and naviculocuneiform dislocation associated with a fracture through the body of the navicular bone [1-3,6-8].

Our case also differs from previously reported injury patterns due to the associated calcaneus fracture. Main and Jowett described lateral dislocations of the midfoot to be associated with cuboid fractures secondary to compression between the calcaneus and metatarsals, or “nutcracker fractures” [9]. Kummer et al. described a patient with calcaneocuboid and naviculocuneiform dislocations with associated avulsion fractures of the medial cuneiform and anterior process of the calcaneus. These injuries were treated with pinning of the calcaneocuboid joint and ORIF of the naviculocuneiform joint with a dorsal spanning plate [3]. Fares et al. reported a patient with dislocation of the calcaneocuboid and naviculocuneiform joints with an impaction fracture of the anterior process of the calcaneus sustained after a crush injury, also treated with pinning of the calcaneocuboid joint and dorsal spanning plate of the naviculocuneiform joint [7]. We describe the utilization of a five-hole 3.5 mm lapidus plate to bridge the calcaneocuboid joint, which was then subsequently removed. This represents a unique approach to the management of this pathology.

Kang et al. outlined a similar case with a fracture of the anterior process and lateral wall of the calcaneus treated with pinning of the naviculocuneiform and calcaneocuboid joints without fixation of the calcaneus fracture. There was residual subluxation ultimately requiring salvage arthrodesis [10]. Our patient also had associated fractures of both the anterior process and lateral wall of the calcaneus from impaction of the cuboid. Treatment required elevation of the cuboid and the articular surface of the calcaneus, followed by fixation of the fractured calcaneus with a K-wire and plate with good outcomes at four and a half months.

## Conclusions

Closed dislocations of the calcaneocuboid and naviculocuneiform joints are extremely rare. They can be associated with fractures of both the navicular bone and calcaneus and can occur after both high- and low-energy injuries. Further reporting of these rare injuries is necessary to guide future management guidelines.

## References

[1] Cheng Y, Yang H, Sun Z, Ni L, Zhang H (2012). A rare midfoot injury pattern: navicular-cuneiform and calcaneal-cuboid fracture-dislocation. J Int Med Res.

[2] Alayed IS (2021). Combined midfoot dislocation involving the naviculocuneiform and calcaneocuboid joints: a case report. Egypt J Hosp Med.

[3] Kummer A, Crevoisier X, Eudier A (2020). Calcaneocuboid and naviculocuneiform dislocation: an unusual injury of the midfoot. Case Rep Orthop.

[4] Wong KP, Tang ZH, Tan GM (2020). Combined open calcaneocuboid, naviculocuneiform and subtalar dislocation: a case report and literature review. Biomedicine (Taipei).

[5] Dhole KP, Bandebuche AR, Marathe NA, Date S, Raj A (2020). An unusual midfoot dislocation involving naviculocuneiform and calcaneocuboid joint following low-energy injury: a case report. J Orthop Case Rep.

[6] Chopra RK, Pushpasekaran N, Palanisamy S, Ravi B (2017). A complex midtarsal dislocation of the foot following a supination abduction injury: a case report. Foot Ankle Online J.

[7] Fares A, Orfeuvre B, Al Ezz MA, Pailhe R (2022). An unusual midfoot injury pattern: navicular-cuneiform and calcaneal-cuboid fracture-dislocation. Trauma Case Rep.

[8] Alhadhoud MA, Alsiri NF, Mohammad DA, Ibrahim A, Aboubakr MK, Abdulghany M, Fathy A (2022). Open fracture dislocation of the calcaneocuboid and naviculocuneiform joints: a case report. Trauma Case Rep.

[9] Main BJ, Jowett RL (1975). Injuries of the midtarsal joint. J Bone Joint Surg Br.

[10] Kang GC, Rikhraj IS (2008). Salvage arthrodesis for fracture-dislocation of the cuneonavicular and calcaneocuboid joints: a case report. J Orthop Surg (Hong Kong).

